# COVID-19 in solid organ transplant recipients after 2 years of pandemic: Outcome and impact of antiviral treatments in a single-center study

**DOI:** 10.3389/frtra.2023.1095225

**Published:** 2023-03-16

**Authors:** Biagio Pinchera, Antonio Riccardo Buonomo, Emilia Trucillo, Stefano Susini, Alessia D’Agostino, Isabella Di Filippo, Anastasia Tanzillo, Riccardo Villari, Rosa Carrano, Roberto Ivan Troisi, Ivan Gentile

**Affiliations:** ^1^ Section of Infectious Diseases, Department of Clinical Medicine and Surgery, University of Naples “Federico II”, Naples, Italy; ^2^ Section of Nephrology, Department of Public Health, University of Naples “Federico II”, Naples, Italy; ^3^ Division of Hepato-Bilio-Pancreatic, Minimally Invasive, Robotic and Transplant Surgery, Department of Clinical Medicine and Surgery, Federico II University Hospital, Naples, Italy

**Keywords:** COVID-19, SARS-CoV-2, solid organ transplant, antiviral, treatment

## Abstract

More than 2 years after the pandemic, the availability of vaccination and the use of monoclonal antibodies and direct antivirals have changed the fate of COVID-19, allowing for a better management of the disease, reducing hospitalization rates, and improving survival. This study aims to describe the outcome of COVID-19 in a cohort of solid organ transplant recipients and the impact of novel antivirals against SARS-CoV-2. We conducted an observational retrospective cohort study. We enrolled solid organ transplant recipients with COVID-19 attending the A.O.U. Federico II of Naples and followed up from January 2022 to July 2022. We enrolled 40 SOTs with COVID-19. Our experience highlights the favorable impact of therapies with antivirals and monoclonal antibodies in the early stages of COVID-19. Interesting data concern the impact of immunosuppressive therapy on COVID-19, in particular the role of Mycophenolate (associated with deterioration to severe COVID-19) and Everolimus (protective for progression to severe disease) needs to be investigated. Our experience also confirms the fundamental role of vaccination and in particular the importance of the booster dose.

## Introduction

More than 2 years after the pandemic, the availability of vaccination and the use of monoclonal antibodies and direct antivirals have changed the fate of COVID-19 (COronaVIrus Disease-19), allowing for a better management of the disease, reducing hospitalization rates, and improving survival. However, some categories of patients, such as immunocompromised patients, and in particular solid organ transplant patients (SOT) constitute a category of patients still at risk for a severe form of the disease (1, 2). In fact, SOTs have a higher risk of COVID-19 related hospitalization, morbidity and mortality (3–5).

Moreover, considering general population of patients with COVID-19, both monoclonal antibodies and antivirals have shown excellent results in reducing both hospitalization and the mortality rate, if administered early after symptoms onset (6–8). However, data regarding the impact of these drugs in SOT recipients with COVID-19 are scarce, because most studies excluded solid organ transplants (9–11). Currently, available data are limited to a few case series describing the use of monoclonal antibodies and antivirals in SOTs (12–15).

This study aims to describe the outcome of COVID-19 in a cohort of solid organ transplant recipients and the impact of novel antivirals against SARS-CoV-2 (Severe Acute Respiratory Syndrome Coronavirus 2).

## Materials and methods

We conducted an observational retrospective cohort study. We enrolled solid organ transplant recipients with COVID-19 attending the A.O.U. Federico II of Naples and followed up from January 2022 to July 2022. Patients underwent rhino-oropharyngeal swabs in presence of symptoms suspected for COVID-19. Diagnosis of COVID-19 was defined as positivity to the rhino-oropharyngeal swab for SARS-CoV-2 RNA research by reverse transcription—polymerase chain reaction (RT-PCR) in presence of at least one typical COVID-19 symptom (fever, malaise, cough, nausea/diarrhea, shortness of breath, headache, nasal stuffiness, anosmia, dysgeusia). To describe the clinical status of COVID-19 we used the NIAID ACTT-1 (National Institute of Allergy and Infectious Diseases Adaptive COVID-19 Treatment Trial-1) Clinical Status Ordinal Scale (16). Based on this score, we classified each patient with the infection into one of eight categories: (1) Not hospitalized, no limitations on activities; (2) Not hospitalized, limitation on activities, and/or requiring home oxygen; (3) Hospitalized, not requiring supplemental oxygen and no longer requires ongoing medical care (if hospitalization extended for infection-control purposes); (4) Hospitalized, not requiring supplemental oxygen; requiring ongoing medical care (COVID-19 related or otherwise); (5) Hospitalized, requiring supplemental oxygen; (6) Hospitalized, on noninvasive ventilation or high-flow oxygen devices; (7) Hospitalized, on invasive mechanical ventilation or ECMO; (8) Death (16).

In addition, we also distinguished patients with a mild-moderate form of COVID-19 from those with a severe form of COVID-19, based on their need of oxygen (a severe form was defined as a NIAID ACTT-1 Clinical Status Ordinal Scale score ≥ 5).

For each patient we evaluated epidemiological and clinical characteristics, laboratory and radiological data, need of hospitalization and access to the ICU (Intensive Care Unit), type of immunosuppressive treatment and the changes of immunosuppression during SARS-CoV-2 infection, treatment for COVID-19 and outcome. For each patient we evaluated SARS-CoV-2 IgG (Roche Diagnostics GmbH, Mannheim, positive threshold >15 BAU/ml) assessed at enrollment, before the infusion of monoclonal antibodies (if any). Data are presented as mean and SD or median and interquartile range (IQR), in case of Gaussian or non-Gaussian distribution, respectively. For correlation analysis, Pearson or Spearman tests were used for data distributed in Gaussian or non-Gaussian fashion, respectively. Continuous variables are compared by Student's *t*-test or Mann-Whitney *U*-Test, as parametric or non-parametric test, respectively. The *p*-value for statistical significance was set at <0.05 for all the tests. A logistic regression model was employed to evaluate risk factors for severe disease evolution. In particular, age, sex, comorbidities and immunosuppressive therapy were assessed and compared. The study was conducted in compliance with the Declaration of Helsinki and the principles of good clinical practice. The study was exempt from approval from an ethics’ board.

## Results

We enrolled 40 SOTs with COVID-19. Anagraphic and clinical features of these patients are reported in Tables 1–3. Of the 40 patients with COVID-19, 11 were severe (27.5%) and 13 needed hospitalization (32.5%) (Tables 1, 2). Of the 13 hospitalized patients, 4 (30.8%) patients died, which gives a mortality rate of 10% (Table 2).

**Table 1:** Characteristics of enrolled patients.

**Age (median, IQR)**	55 (27–77)
**Gender**	
M	28 (70%)
F	12 (30%)
**COVID-19**	
Mild-moderate	29 (72%)
Severe	11 (28%)
**Symptoms**	
Fever	21 (52.5%)
Malaise	14 (35%)
Cough	17 (42.5%)
Nausea/diarrhea	6 (15%)
Shortness of breath	10 (25%)
Headache	6 (15%)
Nasal stuffiness	5 (12.5%)
Anosmia	2 (5%)
Dysgeusia	2 (5%)
**Comorbidities**	
Hypertension	28 (70%)
Dyslipidemia	15 (37.5%)
Diabetes	11 (27.5%)
Obesity	5 (12.5%)
Cardiovascular disease	3 (7.5%)
**Type of transplant**	
Kidney transplant	35 (87.5%)
Kidney-pancreas transplant	2 (5%)
Liver transplant	2 (5%)
Heart transplant	1 (2.5%)
Time from transplant (months), mean (IQR)	20 (3–252)
**Immunosuppressive therapy at diagnosis**	
Cyclosporine-Everolimus-Steroids	2 (5%)
Cyclosporine-Mycophenolate-Steroids	4 (10%)
Cyclosporine-Mycophenolate	1 (2.5%)
Tacrolimus-Mycophenolate-Steroids	13 (32.5%)
Tacrolimus-Everolimus-Steroids	6 (15%)
Tacrolimus-Mycophenolate	6 (15%)
Tacrolimus-Steroids	7 (17.5%)
Tacrolimus	1 (2.5%)
**COVID-19 vaccination** *	
Yes	29 (72%)
**Ig anti-SARS-CoV-2 titer post vaccination (BAU/ml)**	
Positive	15 (52%)
**Laboratory tests**	**TOE** **
**WBC (cell/µl; median, IQR)**	5,840 (1,250–13,420)
**Neutrophil count (cell/µl; median, IQR)**	4,710 (1,490–10,880)
**Lymphocyte count (cell/µl; median, IQR)**	780 (137–1,920)
**RBC (cell/µl; median, IQR)**	4,120,000 (2,780,000–5,320,000)
**PLT (cell/µl; median, IQR)**	259,000 (79,000–645,000)
**D-dimer (ng/ml; median, IQR)**	734 (270–1,802)
**Fibrinogenemia (mg/dl; median, IQR)**	479 (221–666)
**CRP (mg/L; median, IQR)**	155 (0–156)
**LDH (U/L; median, IQR)**	295 (171–670)

* At least two doses.

** TOE, time of enrolment; IQR, interquartile range; WBC, white blood cells; PLT, platelets; CRP, C-reactive protein; LDH, lactic dehydrogenase.

**Table 2:** NIAID ACTT-1 clinical status ordinal scale of enrolled patients.

Clinical status ordinal scale	Clinical status description for assessment	SOTs with COVID-19 n. 40 (%)
1	Not hospitalized, no limitations on activities	15 (37.5%)
2	Not hospitalized, limitation on activities, and/or requiring home oxygen	12 (30%)
3	Hospitalized, not requiring supplemental oxygen and no longer requires ongoing medical care	0
4	Hospitalized, not requiring supplemental oxygen; requiring ongoing medical care (COVID-19 related or otherwise)	2 (5%)
5	Hospitalized, requiring supplemental oxygen	5 (12.5%)
6	Hospitalized, on noninvasive ventilation or high-flow oxygen devices	1 (2.5%)
7	Hospitalized, on invasive mechanical ventilation or ECMO	1 (2.5%)
8	Death	4 (10%)

**Table 3:** Mild-moderate COVID-19 vs. severe COVID-19 in SOTs.

	Total	Mild-moderate COVID-19	Severe COVID-19	*p*-value
	*n* = 40 (%)	*n* = 29 (%)	*n* = 11 (%)	
**Age (median, IQR)**	55 (27–77)	49 (27–69)	58 (46–77)	***0.084***
**Sex**				
* Male*	28 (70)	20 (69)	8 (73)	*0.210*
* Female*	12 (30)	9 (31)	3 (27)	* *
**Comorbidity**				
* Obesity BMI > 30Kg/m^2^*	5 (12.5)	1 (3.4)	4 (36.3)	***0.039***
* Cardiovascular disease*	3 (7.5)	1 (3.4)	2 (18.1)	*0.150*
* Diabetes mellitus*	11 (27.5)	4 (13.8)	7 (63.6)	*0.270*
* Hypertension*	28 (70)	19 (65.5)	9 (81.8)	*0.130*
* Dyslipidemia*	15 (37.5)	7 (24.1)	8 (72.7)	*0.120*
**Laboratory at time of enrollment**				
* CPR > 5 mg/dl*	27 (67.5)	16 (55.1)	11 (100)	***0*** ***.*** ***043***
* Lymphopenia* *	16 (40)	6 (20.7)	10 (91)	***0*** ***.*** ***047***
**Vaccination status**				
* Booster dose*	17 (42.5)	15 (43.8)	2 (18.1)	***0*** ***.*** ***038***
* Fully vaccinated (2 doses)*	12 (30)	7 (16.7)	5 (45.5)	*0* *.* *120*
* Not Vaccinated* ^§^	11 (27.5)	7 (39.6)	4 (36.4)	***0*** ***.*** ***045***
**Ig anti-SARS-CoV-2 titer**				
* Negative*	14 (48)	10 (45.5)	4 (57)	*0* *.* *280*
**Immunosuppressive therapy at diagnosis**				
* Cyclosporine containing regimens* **	7 (17.5)	4 (13.8)	3 (27.3)	*0* *.* *140*
* Tacrolimus containing regimens* **	33 (82,5)	25 (86)	8 (72.7)	*0* *.* *230*
* Mycophenolate containing regimens* **	24 (60)	14 (48.2)	10 (91)	***0*** ***.*** ***039***
* Everolimus containing regimens* **	8 (20)	8 (27.6)	0	***0*** ***.*** ***031***
* Steroids containing regimens* **	32 (80)	23 (79.3)	9 (81.8)	*0* *.* *310*
**Treatments for COVID-19**				
* Steroid*	11 (27.5)	0°	11 (100)	* *
* Low molecular weight heparin*	13 (32.5)	2 (7)	11 (100)	* *
* Remdesivir*	5 (12.5)	0°	5 (45.5)	* *
* Remdesivir early stage treatment*	3 (7.5)	3 (10.3)	0	***0*** ***.*** ***044***
* Molnupiravir*	7 (17.5)	7 (24)	0	***0*** ***.*** ***035***
* Casirivimab/Imdevimab 600 mg/600 mg*	2 (5)	2 (6.9)	0	***0*** ***.*** ***057***
* Casirivimab/Imdevimab 1.200 mg/1.200 mg*	1 (2.5)	0°	1 (9)	* *
* Sotrovimab*	17 (42.5)	14 (48.2)	3 (27)	***0*** ***.*** ***038***
* Tocilizumab*	1 (2.5)	0°	1 (9)	* *
* Baricitinib*	1 (2.5)	0°	1 (9)	* *
**Hospitalization rate**	11 (27.5)	0	11 (100)	* *
**Outcome**				
* Clinical Healing*	36 (90)	29 (100)	7 (64)	* *
* Death*	4 (10)	0	4 (36)	* *

IQR, interquartile range; BMI, body mass index; CPR, C-reaction protein.

Statistically significant values are highlighted in bold and italics.

* Lymphopenia described as lymphocyte < 800cell/mm^2^.

^§^ Including people who received one dose.

** vs. other treatments.

° Therapy not indicated for mild-moderate COVID-19.

Regarding comorbidities, most of patients (80%) with obesity (defined as a BMI >30 Kg/m^2^) developed a severe form of the disease (*OR 1.5, 95, CI: (1.3–1.9 obese B vs. non obese patients; p = 0.039*) (Table 3).

Most patients with severe COVID-19 showed a CRP > 5 mg/dl (100%) and/or Lymphopenia described as lymphocyte < 800 cells/mm^2^ (91%) (*OR for severe COVID-19: 1.4, 95, CI: (1.1–1.6) CPR > 5 mg/dl vs. CRP *>* 5 mg/dl; p = 0.043; 1.3, 95, CI: (1.1–2) Lymphopenia vs. non-lymphopenia; p = 0.047*) (Table 3). Of the enrolled patients, only 13 patients performed high-resolution lung computed tomography (HRCT); in particular, only hospitalized patients performed HRCT. The severity score index, as proposed by Chung et al. (17) was used for the analysis of each individual HRCT. The 13 patients had a median severity score index equal to 12/20 (median, IQR 7–14) as proposed by Chung et al. (17).

Most patients (82%) with severe COVID-19 were found to be unvaccinated or vaccinated with only two doses [*OR for severe COVID-.19: 1.5, 95, CI: (1.3–1.8) unvaccinated or 2 doses vs. three-dose vaccinated patients; p = 0.045*].

All 40 patients were undergoing immunosuppressive therapy at the time of enrollment and data concerning the different immunosuppressive regimens also in relation to clinical presentation and outcome are given in Tables 1, 3. Of the 11 patients with severe COVID-19, about 90% took mycophenolate [*OR for severe COVID-19: 1.8, 95, CI: (1.3–2.1) Mycophenolate-based vs. other non-mycophenolate-based regimens; p = 0.039*], while no patients receiving everolimus developed a severe form of the disease (Table 3).

Regarding the antiviral therapy in the early stages of the disease, in particular regarding the use of early Remdesivir and Molnupiravir in mild-moderate COVID-19, it was observed that no patient who underwent treatments showed a deterioration towards a severe disease (Table 3).

About the use of monoclonal antibodies, in particular Casirivimab/Imdevimab 600 mg/600 mg and Sotrovimab in mild-moderate COVID-19, it was observed that only 3 out of 19 patients who were treated with monoclonal antibodies in the early stages of the disease developed then a severe form of the disease (*OR for severe COVID-19: 0.9, 95, CI: (0.8–1.1) Casirivimab/Imdevimab treatment vs. no Casirivimab/Imdevimab treatment; p = 0.057; 0.7, 95, CI: (0.5–0.9) Sotrovimab treatment vs. no Sotrovimab treatment; p = 0.038*) (Table 3).

Pooling patients who underwent all antiviral treatments in the early stages of the disease, (both direct antivirals and monoclonal antibodies), only 3 of these 29 patients (10%) developed a severe disease, compared with 8 of 11 (72%) who did not undergo such a treatment [*OR for severe COVID-19: 0.7, 95, CI: (0.5–0.8) antiviral early treatment vs. no early antiviral treatment; p = 0.036*]. At multivariate analysis, the use of early antiviral treatments was confirmed to be independently associated with a reduced risk of developing severe disease [*OR for severe COVID-19: 0.8, 95, CI: (0.5–0.9); p = 0.041]* Table 4.

**Table 4:** Multivariate regression analysis for severe COVID-19 in SOTs.

	OR	95% CI	*p*-value
**Age**	1.4	0.9–1.7	*0.110*
**Male sex**	1.2	0.7–1.4	*0.140*
**Comorbidity**			
* Obesity BMI > 30Kg/m^2^*	1.5	0.9–1.7	*0.115*
* Cardiovascular disease*	1.2	0.7–1.5	*0.130*
* Diabetes mellitus*	1.4	0.8–1.9	*0.210*
* Hypertension*	1.2	0.6–1.3	*0.150*
* Dyslipidemia*	1.3	0.8–1.5	*0.138*
**Laboratory at time of enrollment**			
* CPR > 5 mg/dl*	1.4	0.9–1.6	*0* *.* *086*
* Lymphopenia* *	1.2	0.7–1.3	*0* *.* *098*
**Vaccination status**			
* Booster dose*	0.8	0.6–1.1	*0* *.* *093*
* Fully vaccinated (2 doses)*	1.2	0.8–1.5	*0* *.* *180*
* Not vaccinated* ^§^	1.4	0.9–1.7	*0* *.* *099*
**Ig anti-SARS-CoV-2 titer**			
* Negative*	1.3	0.8–1.5	*0* *.* *105*
***Immunosuppressive therapy at diagnosis***			
* Cyclosporine containing regimens* **	1.2	0.7–1.3	*0* *.* *110*
* Tacrolimus containing regimens* **	1.1	0.9–1.5	*0* *.* *130*
* Mycophenolate containing regimens* **	1.5	1.2–1.8	***0*** ***.*** ***038***
* Everolimus containing regimens* **	0.8	0.6–0.9	***0*** ***.*** ***046***
* Steroids containing regimens* **	1.3	0.8–1.4	*0* *.* *190*
***Early treatments for COVID-19***	0.8	0.5–0.9	***0*** ***.*** ***041***

OR, odds ratio; BMI, body mass index; CPR, C-reaction protein.

Statistically significant values are highlighted in bold and italics.

* Lymphopenia described as lymphocyte < 800cell/mm^2^.

** vs. other treatments.

^§^ Including people who received one dose.

## Discussion

First of all, our study shows that after 2 years of pandemic the hospitalization rate and the mortality rate of SOTs with COVID-19 have decreased. In fact, while at the beginning of the pandemic it was estimated a hospitalization rate of about 32%–100% (18–20) and a mortality rate of about 13%–30% (21), after 2 years, from our study there was a hospitalization rate of about 27% and a mortality rate of about 10%. These data are in agreement with those reported by Heldman and colleagues, who showed that hospitalization rate and the mortality rate in SOTs were gradually decreasing (22). Probably, this change is due to the higher awareness and higher ability to manage COVID-19, thanks to the availability of various therapeutic resources that have occurred over time. However, although reduced, the hospitalization rate and the mortality rate remain quite high in this category of patients.

Although SOTs are already by themselves a category at risk of developing severe COVID-19, obesity with BMI >30 Kg/m^2^ was confirmed as a risk factor for severe disease [OR for obesity BMI > 30 Kg/m^2^: 1.5, 95, CI: (1.3–1.9) severe COVID-19 vs. mild-moderate COVID-19; *p* = 0.039] (Table 3) (23). Instead, the other comorbidities did not appear to be a severe risk factor for COVID-19, confirming what was reported by Opsomer et al. (24).

At the same time, an elevated CRP (CPR > 5 mg/dl) and lymphopenia (lymphopenia described as lymphocyte < 800 cell/mm^2^) were confirmed as predictors of a possible evolution of the disease in a severe form (25, 26).

The type of SOT and the time after transplantation did not constitute a risk factor for severe disease (OR 1.1, 95%, CI: 0.7–2.1], *p* = 0.210 and OR 1.3, 95%, CI: 0.9–1.5], *p* = 0.130). These data were comparable to that is reported by Pereira et al. and Søfteland et al. (27, 28).

Regarding vaccination, patients not vaccinated for COVID-19 showed a higher risk of developing a severe form of COVID-19 [OR for not vaccinated: 1.5, 95, CI: (1.3–1.8) severe COVID-19 vs. mild-moderate COVID-19; *p* = 0.045] (Table 3). These data confirmed what has already been reported in the literature, regarding the fact that vaccinated SOTs have a lower risk of acquiring the infection and a lower risk of developing COVID-19 than unvaccinated SOTs (15% vs. 20%–30% and 11% vs. 35%–40%) (21, 29). Moreover, in our study, booster dose represented a protective factor for the development of a severe form of the disease [OR for booster dose: 0.8, 95, CI: (0.6–0.9) severe COVID-19 vs. mild-moderate COVID-19; *p* = 0.038] (Table 3). These data are in agreement to what has been reported in the study conducted by Marinaki et al. (30), underlining the importance of the booster dose and the need to vaccinate this category of patients with multiple doses (31, 32).

In our study, the use of Mycophenolate was a risk factor for the development of a severe form of COVID-19 [OR for Mycophenolate-based vs. other non-mycophenolate-based regimens: 1.8, 95, CI: (1.3–2.1) severe COVID-19 vs. mild-moderate COVID-19; *p* = 0.039], while the presence of Everolimus in the immunosuppressive regimen represented a protective factor against severe COVID-19 [OR for Everolimus-based vs. other non-everolimus-based regimens: 0.7, 95, CI: (0.5–0.8) severe COVID-19 vs. mild-moderate COVID-19; *p* = 0.031]. Regarding the use of mycophenolate, our data confirmed what was reported by Colmenero et al. and Requião-Moura (33, 34). Indeed, Colmenero et al. showed that severe COVID-19 was independently associated with mycophenolate-containing regimens [OR 3.94 (95% CI 1.59–9.74); *p* = 0.003] (33). However, other studies conducted on SOTs with COVID-19, in particular, Webb et al. and Pereira et al., did not confirm such data (27, 35).

The finding of the favorable impact of mTOR inhibitors confirmed what was reported by Heldman et al. (22) and what reported from our previous experience, in which the patients who received mTOR inhibitors, as part of their immunosuppressive therapy, compared to other regimens had a lower chance of developing a moderate or severe form of the disease [OR = 0.8, 95, CI: (0.21–0.92), *p* = 0.041] (36). However, our own data are not in agreement to what has been reported by Genuardi et al. (37). In this study, in fact, -inhibitor use was shown to be an independent risk factor for severe COVID-19 [OR 6.80 (95% CI 1.30–41.00), *p* = 0.026]. However, it is noteworthy that Genuardi's study included only heart transplant patients and only 15 were patients who practiced therapy with mTOR inhibitors (37). At the same time, there are studies that did not find any role of mTOR inhibitors in the evolution of COVID-19 (38, 39).

Calcineurin inhibitors were confirmed as non-risk factors for disease severity, as reported in the literature (33, 37).

In consideration of the few data in the literature concerning the use of antivirals in SOTs, our study showed the impact of antivirals, in particular it highlighted how the use of antivirals in the early stages of mild-moderate disease was associated with a lower risk of developing severe disease (OR for early Remdesivir vs. no early Remdesivir: 0.8, 95, CI: (0.6–0.9) severe COVID-19 vs. mild-moderate COVID-19; *p* = 0.044; OR for Molnupiravir vs. no Molnupiravir: 0.7, 95, CI: (0.4–0.9) severe COVID-19 vs. mild-moderate COVID-19; *p* = 0.035) (Table 3). We underline that in this cohort of patients we considered only the use of early Remdesivir and Molnupiravir, due to the potential drug interactions nirmatrelvir/ritonavir with immunosuppressive therapy and others concomitant therapies. Our data were confirmed by several studies, in fact the data regarding early Remdesivir treatment in SOT agreed with the study by Colaneri et al., in which treatment with early Remdesivir was shown to prevent severe COVID-19 [HR: 0.05; C.I. (0.00–0.65), *p* = 0.01i] (14). At the same time Villamarín et al. showed how the use of Molnupiravir in SOTs could reduce the risk of hospitalization and mortality, confirming our data (40). However, we underline that currently data on the use of antivirals in SOTs are scarce and scanty so far.

Regarding monoclonal antibodies, in our study the use of Casirivimab/Imdevimab (600 mg/600 mg) and Sotrovimab (500 mg) in the early stages of the disease was also associated with a lower risk of developing severe disease. However, the use of Casirivimab/Imdevimab had no significant impact on outcome [OR for severe COVID-19 vs. mild-moderate COVID-19: 0.9, 95, CI: (0.8–1.1) Casirivimab/Imdevimab 600 mg/600 mg vs. no Casirivimab/Imdevimab 600 mg/600 mg; *p* = 0.057] and this is probably attributable to the small number of individuals considered (n. 3). In contrast, the use of Sotrovimab was associated with a lower risk of developing severe disease [OR for severe COVID-19 vs. mild-moderate COVID-19: 0.7, 95, CI: (0.5–0.9) Sotrovimab vs. no Sotrovimab:; *p* = 0.038]. Our data agree with what has been already reported by Gueguen et al. which showed that SOTs subjected to monoclonals had a lower hospitalization rate (35% vs. 49.7%, *p* = 0.032) and a lower mortality (1.25% vs. 11.6%, *p* = 0.005) compared to those who did not practice treatment with monoclonals (41, 42). In the study of Headvat J et al., patients who received Sotrovimab (*N* = 51) experienced a lower rate of 30-day hospitalization or death as compared to those who received no specific treatment (*N* = 75) (*p* = 0.009) (9). Similar results were also reported by Cochran et al. and also from our previous experience, which showed that treatment with Sotrovimab significantly was associated with a low risk of developing a severe disease (12, 43). Regarding the choice of the different monoclonal antibodies, we underline that this should be related to the circulating variants. In fact the advent of the Omicron variant, and in particular of the BA.1 variant, led to a main use of sotrovimab, due to the reduced activity and efficacy of the other monoclonal antibodies against this variant (43, 44). Subsequently, with the advent of the BA.2 variant led to a reduction of use of sotrovimab due to its reduced activity towards this new variant (44).

Our study shows a significant and favorable impact of antiviral therapies used in the early stages of COVID-19 in SOTs, highlighting how direct antiviral therapies and monoclonal antibodies have changed the evolution and fate of the disease. Our study underlines the importance of timely intervention with these therapies in the early stages of COVID-19, as they allow us to reduce the risk of severe disease evolution.

We acknowledge that our study has several limitations, in particular they are: small number of the sample, single-center retrospective observational study, the absence of a control group and the consideration of only patients with COVID-19 and not all SOTs with SARS-CoV-2 infection.

The main strength of our study is the topical update of the data in a time of availability of several weapons against COVID-19, such as antivirals and monoclonal antibodies, allowing a current view of disease in SOTs.

## Conclusion

Our study, albeit monocentric and with a small number of subjects considered, offers an overview of what the current status of COVID-19 in SOTs at about 2 years after the start of the pandemic. In particular, our real-life experience highlights the favorable impact of therapies with antivirals and monoclonal antibodies in the early stages of COVID-19. However, further studies are needed to investigate and confirm these data. Interesting data concern the impact of immunosuppressive therapy on COVID-19, in particular the role of Mycophenolate (associated with deterioration to severe COVID-19) and Everolimus (protective for progression to severe disease) needs to be investigated. Our experience also confirms the fundamental role of vaccination and in particular the importance of the booster dose. Despite all these weapons, our study underlines that SOTs are still to be considered a category of patients at high risk for developing severe COVID-19.

## Data Availability

The raw data supporting the conclusions of this article will be made available by the authors, without undue reservation.

## Federico II COVID team

Luigi Ametrano, Francesco Beguinot, Giuseppe Castaldo, Letizia Cattaneo, Maria Carmela Domenica Conte, Mariarosaria Cotugno, Alessia d’Agostino, Giovanni Di Filippo, Isabella Di Filippo, Antonio Di Fusco, Nunzia Esposito, Mariarosaria Faella, Lidia Festa, Maria Foggia, Maria Elisabetta Forte, Ludovica Fusco, Antonella Gallicchio, Ivan Gentile, Agnese Giaccone, Carmela Iervolino, Antonio Iuliano, Amedeo Lanzardo, Federica Licciardi, Matteo Lorito, Simona Mercinelli, Fulvio Minervini, Giuseppina Muto, Mariano Nobile, Biagio Pinchera, Giuseppe Portella, Laura Reynaud, Alessia Sardanelli, Marina Sarno, Nicola Schiano Moriello, Maria Michela Scirocco, Fabrizio Scordino, Riccardo Scotto, Stefano Mario Susini, Anastasia Tanzillo, Grazia Tosone, Maria Triassi, Emilia Trucillo, Annapaola Truono, Ilaria Vecchietti, Giulio Viceconte, Riccardo Villari, Emanuela Zappulo, Irene Zotta and Giulia Zumbo.
